# *Candida albicans* and *Candida dubliniensis* Show Different Trailing Effect Patterns When Exposed to Echinocandins and Azoles

**DOI:** 10.3389/fmicb.2020.01286

**Published:** 2020-06-16

**Authors:** Rania Ayadi, Emilie Sitterlé, Christophe d’Enfert, Eric Dannaoui, Marie-Elisabeth Bougnoux

**Affiliations:** ^1^Unité de Parasitologie-Mycologie, Service de Microbiologie, Faculté de Médecine, APHP, Hôpital Européen Georges Pompidou, Université Paris-Descartes, Paris, France; ^2^Unité de Parasitologie-Mycologie, Service de Microbiologie, Faculté de Médecine, APHP, Hôpital Necker Enfants-Malades, Université Paris-Descartes, Paris, France; ^3^Unité Biologie et Pathogénicité Fongiques, Institut Pasteur, USC 2019 INRA, Paris, France; ^4^Dynamyc Research Group, Paris Est Créteil University (UPEC, EnvA), Créteil, France

**Keywords:** *C. albicans*, *C. dubliniensis*, trailing, EUCAST, antifungal susceptibility testing, Etest, echinocandins

## Abstract

When *Candida albicans* and *Candida dubliniensis* isolates were tested for susceptibility to fluconazole and echinocandins using either EUCAST or Etest methods, differential patterns of growth were observed, independently of the methods used. For *C. albicans*, a trailing phenomenon (incomplete growth inhibition at supra-MICs) was observed with fluconazole in 90% and 93.3% for EUCAST and Etest, respectively, but not with echinocandins (<7% for EUCAST and 0% for Etest). In contrast, for *C. dubliniensis*, a trailing phenomenon was very rarely observed with fluconazole (20% for EUCAST and 0% for Etest), while the opposite pattern was observed with echinocandins (>50% for EUCAST and >86% for Etest). This suggests that the pathways involved in the trailing effect might be different between these two related species. Furthermore, clinical microbiologists must be aware of these species-specific patterns for a reliable MIC determination.

## Introduction

*Candida dubliniensis* is a commensal of the oral cavity but can also colonize other anatomical sites (Loreto et al., 2010). Furthermore, it can cause oropharyngeal candidiasis, mainly in HIV-positive patients (Sullivan et al., 2005; Loreto et al., 2010), and invasive candidiasis (Pfaller et al., 2014a). *C. dubliniensis* is closely related to *Candida albicans* (Sullivan et al., 2005). *C. albicans* and *C. dubliniensis* share the same *in vitro* antifungal susceptibility patterns (Pfaller et al., 1999; Lortholary et al., 2007). The clinical breakpoints (CBs) and Epidemiological Cut-Off values (ECOFFs), determined by CLSI/EUCAST reference methods or commercial methods are similar (Pfaller et al., 2014b; Espinel-Ingroff et al., 2019^1^). Acquired resistance to antifungal has been reported in *C. dubliniensis* just as in *C. albicans* (Perea et al., 2002; Coleman et al., 2010; Prigent et al., 2017), but less frequently (Pfaller et al., 1999). Until recently, identification of *C. dubliniensis* was difficult in the clinical laboratories because it shares C. *albicans* phenotypic characteristics (Sullivan et al., 2005; Loreto et al., 2010). Things changed with the use of Matrix-Assisted Laser Desorption Ionization–Time of Flight (MALDI-TOF), which can easily differentiate the two species (Roberts et al., 2016). There are some striking differences between *C. albicans* and *C. dubliniensis*. First, their ecological niches are different. Unlike *C. albicans*, *C. dubliniensis* is uncommon in the gastro-intestinal tract of healthy individuals, but for the oral cavity (Strati et al., 2016). Second, there are major difference in term of pathogenicity, *C. albicans* being more pathogenic (Moran et al., 2012).

Recently, we also observed differential growth patterns between *C. albicans* and *C. dubliniensis* when they are tested by Etest for susceptibility to fluconazole and echinocandins. With *C. dubliniensis*, a trailing effect (incomplete growth inhibition at supra-MICs) was observed with echinocandins but not with fluconazole, while for *C. albicans* the opposite pattern was seen. To further assess these differences between the two species, we analyzed, here, the growing patterns of a large panel of clinical isolates of the two species when tested against fluconazole and two echinocandins by the reference EUCAST technique and by Etest.

## Materials and Methods

To study the growth of *C. albicans* and *C. dubliniensis* in the presence of fluconazole, caspofungin or micafungin, a total number of 30 *C. albicans* and 30 *C. dubliniensis* clinical epidemiologically not related isolates were selected from the mycology’s laboratories of Necker and Georges Pompidou University hospitals. None of these strains were known to be resistant to echinocandins or azoles.

These 60 isolates were tested using two different methods: the EUCAST EDef 7.3 microdilution procedure and the Etest agar diffusion method (Biomérieux, Marcy-l’Étoile, France). The yeasts were first cultured on BBL CHROMagar Candida plate (Becton Dickinson GmbH, Heidelberg, France) for primary identification and then identified by MALDI-TOF spectrometry (microflex LT, Bruker Daltonik GmbH, Germany).

Antifungal susceptibility testing by EUCAST was performed according to the reference method (Arendrup et al., 2017) in RPMI 1640 medium buffered to pH 7.0 with morpholino-propanesulfonic acid (MOPS) and supplemented with glucose to a final concentration of 2%. The final inoculum was 0.5–2.5 10^5^ CFU/ml. *Candida krusei* ATCC 6258 and *Candida parapsilosis* ATCC 22019 isolates were used as quality control strains. Spectrophotometric reading was performed after incubation at 35°C, and MICs were determined with a 50% inhibition endpoint. The percentage of growth was determined compared to the drug-free control. The trailing effect was defined as a reduced but persistent (≥5%) growth (G) above the MIC of the isolate, at least in four consecutive wells. Consequently, two groups of trailing effect were defined depending on G, as previously described (Rueda et al., 2017): moderate trailing for G between 5 and 25%, strong trailing when G is >25 but <50%,

Antifungal susceptibility testing by Etest was performed according to manufacturer’s instructions (Biomérieux, 2013). The presence of microcolonies in the ellipse of inhibition after incubation at 35°C defined the trailing effect and was not indicative of resistance. Due to the subjective nature of the visual reading, the level of the trailing effect was not categorized by the Etest method.

For both techniques (EUCAST, 2019) reading was performed at 24 and 48 h. Indeed, for Etest the period incubation depends on the growth and could be 24 h and/or 48 h. Moreover, it is recommended to read at 48 h for detection of some phenomenon such as heteroresistance. For these reasons, Etest reading is often performed both at 24 and 48 h. Therefore, we also determined MICs by EUCAST at 24 h and 48 h.

## Results

With Etest, MIC values at 24 h determined for fluconazole, micafungin, and caspofungin for *C. albicans* ranged from 0.06 to 0.25 μg/ml [Geometric mean (GM) = 0.14 μg/ml], from 0.008 to 0.016 (GM = 0.009 μg/ml), and from 0.03 to 0.25 μg/ml (GM = 0.09 μg/ml), respectively, while for *C. dubliniensis* MICs ranged from 0.03 to 0.5 (GM = 0.09 μg/ml), from 0.008 to 0.016 (GM = 0.01 μg/ml), and from 0.03 to 0.25 (GM = 0.12 μg/ml), respectively. MIC distributions determined by Etest at both 24 h and 48 h are presented in Table 1.

**TABLE 1:** MIC distribution of fluconazole, caspofungin and micafungin against *C. albicans* and *C. dubliniensis* by EUCAST and Etest methods.

**Antifungal**													
				**Concentration (ug/ml)**									
**Species**	**Method**		**ATF**	**0.008**	**0.015**	**0.03**	**0.06**	**0.125**	**0.25**	**0.5**	**1**	**2**	**4**
*C. albicans*	EUCAST	24 h	FCZ					14	14	2			
*C. dubliniensis*								23	5	1		1	
*C. albicans*	EUCAST	24 h	CAS					8	21	1			
*C. dubliniensis*								8	16	6			
*C. albicans*	EUCAST	24 h	MIC		30								
*C. dubliniensis*					30								
*C. albicans*	EUCAST	48 h	FCZ					13	17				
*C. dubliniensis*								22	4	1		1	
*C. albicans*	EUCAST	48 h	CAS					5	21	4			
*C. dubliniensis*								6	11	12	1		
*C. albicans*	EUCAST	48 h	MIC		26	3	1						
*C. dubliniensis*					26	3	1						
*C. albicans*	Etest	24 h	FCZ				2	20	8				
*C. dubliniensis*						2	13	12	2	1			
*C. albicans*	Etest	24 h	CAS			3	9	17	1				
*C. dubliniensis*						1	3	24	2				
*C. albicans*	Etest	24 h	MIC	25	5								
*C. dubliniensis*				15	15								
*C. albicans*	Etest	48 h	FCZ				2	15	13				
*C. dubliniensis*						1	13	13	1	2			
*C. albicans*	Etest	48 h	CAS				9	15	6				
*C. dubliniensis*							3	18	9				
*C. albicans*	Etest	48 h	MIC	20	10								
*C. dubliniensis*				6	21	3							

**FIGURE 1:**
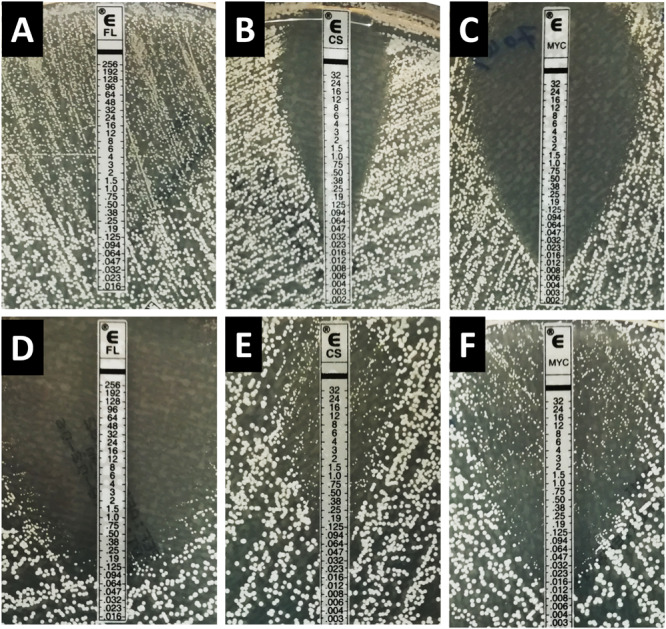
Inhibition pattern by Etest for fluconazole, caspofungin, and micafungin for the strain *C. albicans* HEGP 7043 **(A–C)** and *C. dubliniensis* HEGP 6443 **(D–F)**. FL, fluconazole; CS, caspofungin; MYC, micafungin.

With fluconazole, a trailing effect was observed with Etest for 93.3% of *C. albicans* isolates while no trailing was seen with the two echinocandins (Table 2 and Figure 1). In contrast, for the *C. dubliniensis* isolates, trailing was not observed with fluconazole, while trailing was observed for echinocandins in 86.7% and 90% of the isolates for micafungin and caspofungin, respectively (Table 2 and Figure 1). In order to confirm that the differential pattern of growth was not related to the susceptibility testing method used, all the strains were also tested by EUCAST (Table 1). MICs determined by EUCAST were within ± 2 log_2_ dilutions of the Etest MICs in >98% of the cases.

**TABLE 2:** Trailing observed for fluconazole, micafungin, and caspofungin against *C. albicans* and *C. dubliniensis* by EUCAST and Etest methods.

**Method**	**Species**	**Trailing**	**% of isolates with trailing**					
			**Fluconazole**		**Micafungin**		**Caspofungin**	
			**24 h**	**48 h**	**24 h**	**48 h**	**24 h**	**48 h**
EUCAST	*C. albicans*	Moderate	20	16.7	0	0	0	0
		Strong	73.3	73.3	0	6.7	0	0
		Total	93.3	90	0	6.7	0	0
	*C. dubliniensis*	Moderate	16.7	6.7	6.7	40	3.3	16.7
		Strong	10	13.3	0	16.7	10	33.3
		Total	26.7	20	6.7	56.7	13.3	50
Etest	*C. albicans*	Total	43.3	93.3	0	0	0	0
	*C. dubliniensis*	Total	0	0	3.3	86.7	6.7	90

**FIGURE 2:**
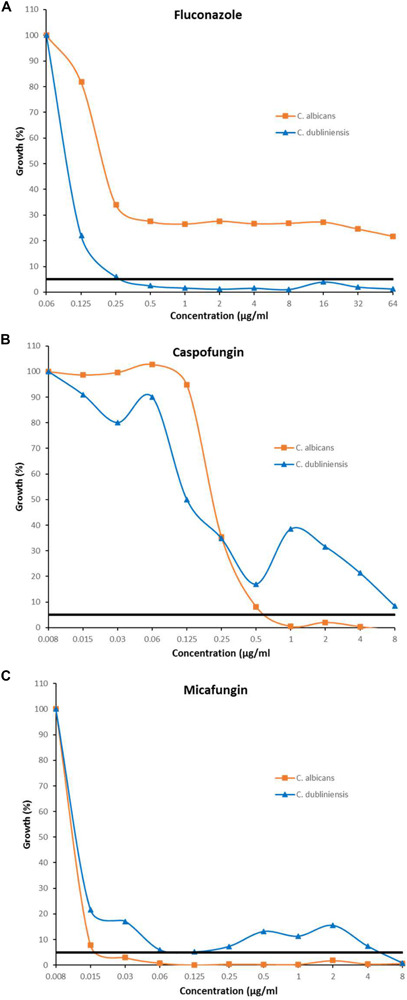
Inhibition pattern by EUCAST in presence of fluconazole **(A)**, caspofungin **(B)**, and micafungin **(C)** for the strains *C. albicans* HEGP 7043 and *C. dubliniensis* HEGP 6409. MICs correspond to the lowest antifungal concentration for which the growth is <50%. Trailing correspond to a growth between 5 and 49%. Black horizontal line represents the 5% growth threshold.

The inhibition patterns obtained with Etest, were also observed by EUCAST for *C. albicans* and *C. dubliniensis* (Table 2). Indeed, for fluconazole, the trailing was seen for *C. albicans* and not for *C. dubliniensis* while the opposite pattern was observed with echinocandins. For *C. albicans*, 90% of the isolates exhibited a trailing effect for fluconazole while only 6.7%, and 0% of the isolates showed a trailing for micafungin, and caspofungin, respectively. For *C. dubliniensis* only 20% of the isolates showed a trailing effect for fluconazole, while 56.7%, and 50% of isolates exhibited a trailing effect for micafungin and caspofungin, respectively (Table 2). All together, these data clearly demonstrated that the trailing is species specific. Figure 2 showed an example of the trailing effect observed for one isolate each of *C. albicans* and *C. dubliniensis* for the three antifungal agents.

The level of trailing effect was always of strong intensity (according to the definition) except for micafungin with *C. dubliniensis* isolates for which a moderate trailing was mostly observed (Table 2).

## Discussion

Although *C. albicans* and *C. dubliniensis* are closely related phylogenetically (Sullivan et al., 2005) and exhibit the same antifungal susceptibility patterns, we found that they behave differently *in vitro* in presence of fluconazole or echinocandins. For *C. albicans*, a trailing phenomenon was observed with fluconazole and not for the echinocandin, while for *C. dubliniensis*, the opposite pattern of growth was seen (i.e., no trailing with fluconazole and trailing with echinocandins). Differential *in vitro* response to echinocandin and azole antifungal agents between *C. albicans* and *C. dubliniensis* has been previously reported by EUCAST and CLSI methods (Arthington-Skaggs et al., 2002; Jacobsen et al., 2007). Our results are in accordance with these previous reports and show that the phenomenon is also present when isolates are tested by a completely different method (i.e., Etest, an agar diffusion method), therefore not dependent on the technique.

Trailing effect of *C. albicans* to fluconazole is well known (Delarze and Sanglard, 2015; Marcos-Zambrano et al., 2016; Rosenberg et al., 2018) and may have a clinical impact (Rosenberg et al., 2018). Here we observed that this effect is not present for the sibling species *C. dubliniensis*. Altogether, these findings could suggest that the pathways involved in the trailing effect may be different in the two species. The comparative study of these two species may help deciphering the mechanisms involved in tolerance in *C. albicans*.

Finally, our findings are important for the clinical microbiology laboratories and clinical care. Indeed, the trailing observed when echinocandins are tested against *C. dubliniensis* should be known and the corresponding strains should not be considered as resistant pattern to these important drugs.

## Data Availability Statement

All datasets generated for this study are included in the article/supplementary material.

## Author Contributions

M-EB, CD’E, and ED designed the study. RA and ES performed the experiments. M-EB, RA, and ED analyzed the data. M-EB and ED wrote the manuscript. All authors listed have approved and corrected the manuscript.

## Disclosure

Outside the present work the authors have the following disclosures: During the past 5 years, ED has received research grants from MSD and Gilead; travel grants from Gilead, MSD, Pfizer, and Astellas, and speaker’s fee from Gilead, MSD, and Astellas. M-EB has received research grants from MSD and Astellas; travel grants from Gilead, MSD, and Astellas, and speaker’s fee from Gilead, MSD, and Astellas.

## Conflict of Interest

The authors declare that the research was conducted in the absence of any commercial or financial relationships that could be construed as a potential conflict of interest.

## Footnotes

1 http://www.eucast.org/astoffungi/clinicalbreakpointsforantifungals/
